# Theoretical Study of Electrostatic Embedding and Properties of a Novel Quinolinone‐Chalcone Crystal and a Comparative Analysis with Dihydroquinolinone Analogs

**DOI:** 10.1002/open.202500527

**Published:** 2026-02-08

**Authors:** Clodoaldo Valverde, Nathália M. Pires, Antônio N. Borges, Daphne C. Fernandes, Vitor S. Duarte, Giulio D. C. D’Oliveira, Jean M. F. Custodio, Caridad N. Pérez, Francisco A. P. Osório, Hamilton B. Napolitano

**Affiliations:** ^1^ Universidade Estadual de Goiás Anápolis Brasil; ^2^ Universidade Paulista – UNIP Goiânia Brasil; ^3^ Pontifícia Universidade Católica de Goiás Goiânia Brasil; ^4^ Instituto de Química Universidade Federal de Goiás Goiânia Brasil; ^5^ Departament of Chemistry and Biochemistry University of Notre Dame Notre Dame Indiana USA; ^6^ Instituto de Física Universidade Federal de Goiás Goiânia Brasil; ^7^ Universidade Evangélica de Goiás Anápolis Brasil

**Keywords:** charge embedding, crystal engineering, dipole moment, nonlinear optics, X‐ray diffraction

## Abstract

In this work, we study the linear and nonlinear optical properties of a novel quinolinone‐chalcone derivative, namely, 4(1H)‐quinolinone‐(E)‐4‐chlorobenzylidene‐4‐chlorophenyl‐phenylsulfonyl with formula C_28_H_19_Cl_2_NO_3_S. Theoretical calculations of the electrical properties of the quinolinone‐chalcone derivative crystal were performed at density functional theory DFT/CAM‐B3LYP/6‐311++G(d, p) level, both in the static and dynamic regimes. To simulate the crystalline environment, an electrostatic iterative charge embedding approach was employed, which revealed a redistribution of electronic density arising from crystalline polarization effects. This approach revealed a significant enhancement in the molecular dipole moment (μ≈5.95D) due to crystal packing effects. The calculated third‐order nonlinear susceptibility at 532 nm was found to be $\chi_{Kerr}^{\left(3\right)}\approx 162.52\times 10^{-22}{\left(m/V\right)}^{2}$, with a highest occupied molecular orbital‐lowest unoccupied molecular orbital gap of 4.14 eV, indicating a good potential for optical switching applications. Future experimental validations via Z‐scan and third‐harmonic generation measurements are proposed to corroborate these theoretical predictions.

## Graphical abstract

This graphical abstract highlights the molecular framework, electronic features, and nonlinear optical response of the quinolinone–chalcone crystal QCCP. The left panel displays the full molecular structure and the electrostatic environment introduced by iterative charge embedding, illustrating the crystal‐packing–induced dipole enhancement observed in the solid state. The central panel summarizes the key electronic mechanism, emphasizing the highest occupied molecular orbital‐lowest unoccupied molecular orbital intramolecular charge‐transfer pathway and the associated energy gap (Δ*E* = 4.14 eV), which underpin the material's optical activity. The right panel presents the principal result of this study: a pronounced third‐order nonlinear susceptibility, χ(3) ≈ 162.52×10^−22^ (m/V)² at 532 nm, positioning QCCP as a viable organic candidate for third‐order photonic applications. Collectively, the panels visualize the relationship between structural organization, electronic distribution, and nonlinear performance.

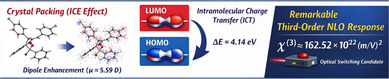

## 1 Introduction

The field of nonlinear optics is getting more important in technological and research development with an increasing number of applications in various areas. The optical response becomes nonlinear [1, 2], when intense laser light interacts with matter; then new phenomena became possible, such as for example, the generating light at new frequencies or having the refractive index change based on how intense the light is. Nonlinear optics gives promising chances for advancements in various areas, as high‐speed optical communications [3, 4, 5], medical images [6], quantum information [7, 8, 9, 10], and making the next generation of photonic devices [11, 12]. Because of this, the hard search for materials that are good for nonlinear optics applications have increased over recent decades [13].

In this area, molecules that have high hyperpolarizability values are very important for these advances to continue [14]. Their role is linked to designing molecules in a careful and strategic way. You choose specific chemical groups so you can change the nonlinear optical response [15, 16, 17, 18]. Chemical principles and theories related to them help explain how this design works. They show how the electron arrangement, the functional groups, and the shape of the molecule affect how it behaves in nonlinear optics. Theoretical and experimental studies are complementary. They guide how to make the materials and check if the nonlinear optical properties are right [19].

Quinolinone derivatives are known for having many kinds of biological activities. Some examples are antibiotic, anticancer, anti‐inflammatory, antidiabetic, antimalarial, and antipsychotic effects [20, 21, 22, 23, 24, 25]. For this reason, they have also started to be a focus in applied chemistry for medicine in recent years [26, 27]. Also, because these quinolinone derivatives are naturally electron‐deficient, their nonlinear optical properties were looked at recently [28]. This work showed that these compounds can be interesting candidates for using in nonlinear optical applications [28, 29, 30, 31]. Recent studies have demonstrated the efficacy of integrating chemical synthesis with advanced in‐silico analyses to design novel optical materials [32, 33]. This multidisciplinary approach allows for a deeper understanding of structure‐property relationships, guiding the development of efficient NLO compounds [34, 35, 36].

In this context, the present work the synthesis [37] and crystallographic characterization for a novel quinolinone derivative, namely, 4(*1H*)‐quinolinone‐(*E*)‐4‐chlorobenzylidene‐4‐chlorophenyl‐phenylsulfonyl (QCCP), with formula C_28_H_19_Cl_2_NO_3_S is described in detail. To characterize the intermolecular and interatomic interactions present in the structure, Hirshfeld surface analysis is utilized, supported by tools such as the 2D‐fingerprints and the *d*_*norm*_ surface. To simulate the polarization effect of the crystalline environment on the asymmetric unit of QCCP, a bulk structure comprising 2,122,416 atoms was constructed through the Iterative Charge Embedding (ICE) method, an electrostatic iterative approach for charge distribution [38, 39, 40, 41]. To explore the influence of different substituents on the nonlinear optical response, this investigation was broadened to include the theoretical analysis of four analogous, previously reported structures: 2‐(4‐ethoxyphenyl)−2,3‐dihydro‐1‐(phenylsulfonyl)quinolin‐4(1H)‐one (QC01), 2‐(4‐methoxyphenyl)−2,3‐dihydro‐1‐(phenylsulfonyl)quinolin‐4(1H)‐one (QC02), 2‐(4‐chlorophenyl)−2,3‐dihydro‐1‐(phenylsulfonyl)quinolin‐4(1H)‐one (QC03), and 2‐(4‐bromophenyl)−2,3‐dihydro‐1‐(phenylsulfonyl)quinolin‐4(1H)‐one (QC04) [42]. These derivatives are structurally analogous, with the key distinction being the nature of the substituent at the para‐position of the phenyl group, which includes electron‐donating groups (ethoxy and methoxy) and electron‐withdrawing halogens (chloro and bromo). While the synthesis and basic characterization of these four derivatives are known, their nonlinear optical properties have not been investigated. This work, therefore, presents the first comprehensive theoretical study of the NLO response for the entire family, employing an advanced electrostatic embedding model. At the DFT/CAM‐B3LYP/6‐311++G(d, p) level, the total dipole moment, the average linear polarizability, the second order hyperpolarizability, and the third‐order nonlinear susceptibility were determined.

## 2 Experimental and Computational Procedures

### 2.1 Synthesis and Crystallographic Characterization

**SCHEME 1:**
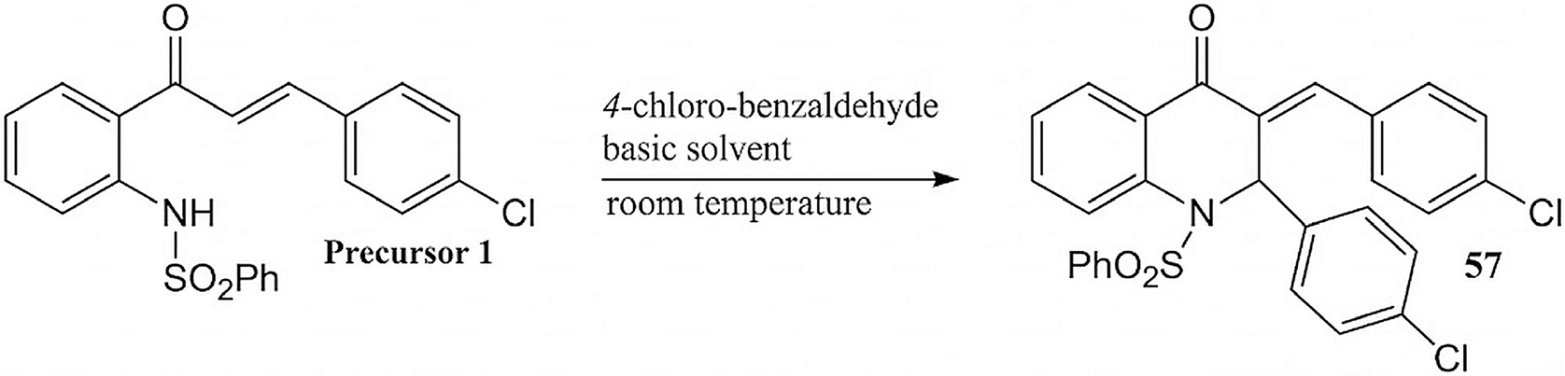
Conditions for the synthesis of chalcone–quinolinone compounds.

Compound QCCP was obtained by reacting 4‐chloro‐benzaldehyde with precursor **1** (chalcone, which was previously synthesized based on the methodology reported by Mirian R. C. de Castro et al. 2016 [43]). The first step of this reaction is the intramolecular addition of the nitrogen of sulfonamide group to *β* carbon of precursor **1**, followed by reaction of *β* carbonyl of the quinolinone formed to the aldehyde by means of Claisen‐Schmidt condensation, yielding the desired compounds. Compound QCCP was synthesized using the methodology reported by Giulio D. C. d’Oliveira et al.*.* 2018 [44] (Scheme 1). Precursor **1** (1.0 mmol) and 4‐chloro‐benzaldehyde (2.0 mmol) were dissolved in 15 mL of basic ethanol (56.1 mg of potassium hydroxide dissolved) and reacted (25°C) for 48hr. The solution was filtered and the precipitate was rinsed with 15 mL of ethanol. The precipitate was dissolved in dichloromethane (10 mL) and this solution was extracted with water. The organic phase was allowed to evaporate slowly, yielding the product.

With the aim of obtaining a single crystal for XRD study, a sample of the compounds were further purified by recristalization by dissolving in dicloromethane and exposing to ethyl ether vapor. (*E*)*‐3‐*(*4‐chlorobenzylidene*)*‐2‐*(*4‐chlorophenyl*)*‐2*,*3‐dihydro‐1‐*(*phenylsulfonyl*)*‐quinolin‐4*(*1H*)*‐one* (**QCCP**)*.* Pale yellow crystalline solid, yield 75.9%, purity of 99.3%, mp 192 – 195°C. ^1^H NMR (CDCl_3_) δ 6.58 (*s*, 1H), δ 7.07 – 7.10 (*m*, 2H), δ 7.10 – 7.12 (*m*, 2H), δ 7.19 – 7.23 (*m*, 2H), δ 7.24 – 7.27 (*m*, 2H), δ 7.31 (*ddd*, *J* 1.14 *Hz*, 7.36 *Hz*, 7.79 *Hz*, 1H), δ 7.34 – 7.37 (*m*, 2H), δ 7.38 – 7.41 (*m*, 2H), δ 7.50 (*tt*, *J* 1.60 *Hz*, 7.80 *Hz*, 1H), δ 7.56 (*s*, 1H), δ 7.56 (*ddd*, *J* 1.68 *Hz*, 7.35 *Hz*, 8.18 *Hz*, 1H), δ 7.71 (*dd*, *J* 0.93 *Hz*, 8.18 *Hz*, 1H), δ 7.88 (*dd*, *J* 1.60 *Hz*, 7.80 *Hz*, 1H); ^13^C NMR (CDCl_3_) δ 59.4, 127.2, 127.5, 128.0, 128.1, 128.3, 129.0, 129.0, 129.3, 129.6, 130.6, 131.2, 131.9, 133.4, 134.6, 134.8, 135.6, 136.7, 137.3, 138.7, 139.2, 182.3; IR 1675 (m), 1609 (m), 1475 (w), 1358 (m), 1302 (m), 1237 (m); HRMS calculated for C_28_H_19_Cl_2_NO_3_S 520.0541, found 520.0569.

The structure of QCCP was determined by a single crystal X‐ray diffraction experiment, with data collected on a Bruker APEX‐II CCD instrument. The QCCP structure was solved with SHEXT‐2014 [45] software by direct methods and refined with SHELXL [46] software by last‐squares minimization, both operated in Olex2 platform [47]. All hydrogens were refined freely. The validation of the crystallographic model was validated with Platon [48]. The crystal data was deposited in the Cambridge Structural Data Centre [49] under deposit code: 2 441 152 and is available free of charge. Examination of geometric parameters of molecules and crystals, as well the generation of Ortep map [50] and figures were performed in Mercury software [51]. Hirshfeld surface was calculated and examined by CrystalExplorer17.5 [52].

### 2.2 Hirshfeld Surface Analysis

The intermolecular interactions found in the QCCP crystal were studied by applying Hirshfeld surface analysis [53]. This surface is defined by Equation (1), wherein the weighting function (ω) is the region where electronic density from molecule ($\rho_{\text{promolecule}}$) overlaps the electronic density from crystal ($\rho_{\text{procrystal}}$), in other words, ω>0.5.

(1) $$\omega \left(r\right)=\frac{\rho_{\text{promolecule}}\left(r\right)}{\rho_{\text{procrystal}}\left(r\right)}$$

This surface has properties that allow a more accurate analysis of intermolecular interactions [54]. The normalized contact distance, known as *d*_norm_, is defined in Equation (2) . This parameter relates the distance from the surface to the closest atom outside (d_e_) and the distance to the closest atom inside (d_i_). Each of these distances is normalized by its corresponding van der Waals distance ($r^{wdV}$) before being combined to give the *d*_norm_ value.

(2) $$d_{\mathrm{norm}}=\frac{d_{i}-r_{i}^{vdW}}{r_{i}^{vdW}}+\frac{d_{e}-r_{e}^{vdW}}{r_{e}^{vdW}}$$

Stronger interactions are highlighted in the *d*_norm_ map as red spots, with intensity proportional of the strength of interaction. The shape index (S) [55] is another useful parameter, employed to analyze the curvature of the surface between molecules. On the shape index map, red areas correspond to convex regions (representing complementary hollows), while blue areas show concave regions (representing bumps). This mapping is helpful for spotting interactions involving *π* systems, as a convex region typically appears around an interacting carbon atom, facing a concave region on the other interacting atom. Equation (3) provides the mathematical definition of the shape index.

(3) $$S=\frac{2}{\pi }\tan^{-1}\left(\frac{k_{1}+k_{2}}{k_{1}-k_{2}}\right)$$

The 2D fingerprint plot [56] was utilized to summarize the contacts around the surface in crystal. It is a diagram of all $d_{e}$ and $d_{i}$ frequencies in crystal and consists in a unique visual representation of all intermolecular interactions of the structure.

### 2.3 Iterative Charge Embedding

In this work, we employ the ICE method to model the influence of the crystalline environment on the electronic properties of a target molecule. ICE is an iterative electrostatic embedding scheme in which the asymmetric unit extracted from the experimental crystal structure data is treated quantum mechanically, while the surrounding environment is simulated via point charges that simulate the electrostatic field generated by neighboring molecules in the lattice.

The bulk of the QCCP crystal structure was constructed as a 17 × 17 × 17 unit cells, each unit cell containing 8 asymmetric units, with 54 atoms per asymmetric unit, resulting in a volumetric system of 2,122,416 atoms. This same approach was applied to the quinolinone‐chalcone derivatives, constructing for each a volumetric system from a 17 x 17 x 17 supercell based on their respective crystallographic data. The bulk for QC01 was formed from a unit cell containing 4 molecules with 50 atoms each, totaling a system of 982,600 atoms. Similarly, the system for QC02 contains 4 molecules per cell with 47 atoms per molecule, resulting in 923,644 total atoms. For QC03, the bulk also has 2 molecules per cell, each with 43 atoms, resulting in 422,518 atoms. The structure of QC04, in turn, features a unit cell with 4 molecules with 43 atoms, which generates a system of 845,036 atoms.

Convergence of the dipole moment serves as the main criterion for the ICE procedure, signaling that molecular charges and interactions with the environment have stabilized. The method starts by obtaining initial partial atomic charges for the central asymmetric unit via the CHELPG method. These charges are then applied to all atoms in the surroundings, treating them as static point charges instead of their original atomic descriptions. Subsequently, the partial atomic charges of the central molecule are recalculated in this electrostatic field (using DFT/CAM‐B3LYP/6‐311++G(d, p)) to get a new set of charges. This new set of charges is then used for the surrounding point charges, and the process cycles. Iteration continues until the embedded central molecule's dipole moment reaches convergence, indicating self‐consistency between the molecule and its electrostatic surroundings.

The method captures both short‐range and long‐range electrostatic effects in a computationally efficient way, without needing full periodic boundary conditions. ICE is especially good at reproducing environment‐sensitive characteristics such as dipole moments, how charges are distributed, and electronic polarization in molecular crystals. The choice of the CAM‐B3LYP functional is supported by recent literature on similar heterocyclic systems. For instance, Arif et al. [57]. demonstrated the reliability of CAM‐B3LYP in predicting nonlinear optical properties of coumarin derivatives, which share structural similarities with quinolinones. Furthermore, recent computational studies by Sarfraz et al. [58]. on pyrimidinone derivatives confirmed that DFT methods accurately describe the electronic structure and reactivity descriptors of nitrogen‐containing heterocycles, reinforcing the validity of our methodological approach.

**FIGURE 1:**
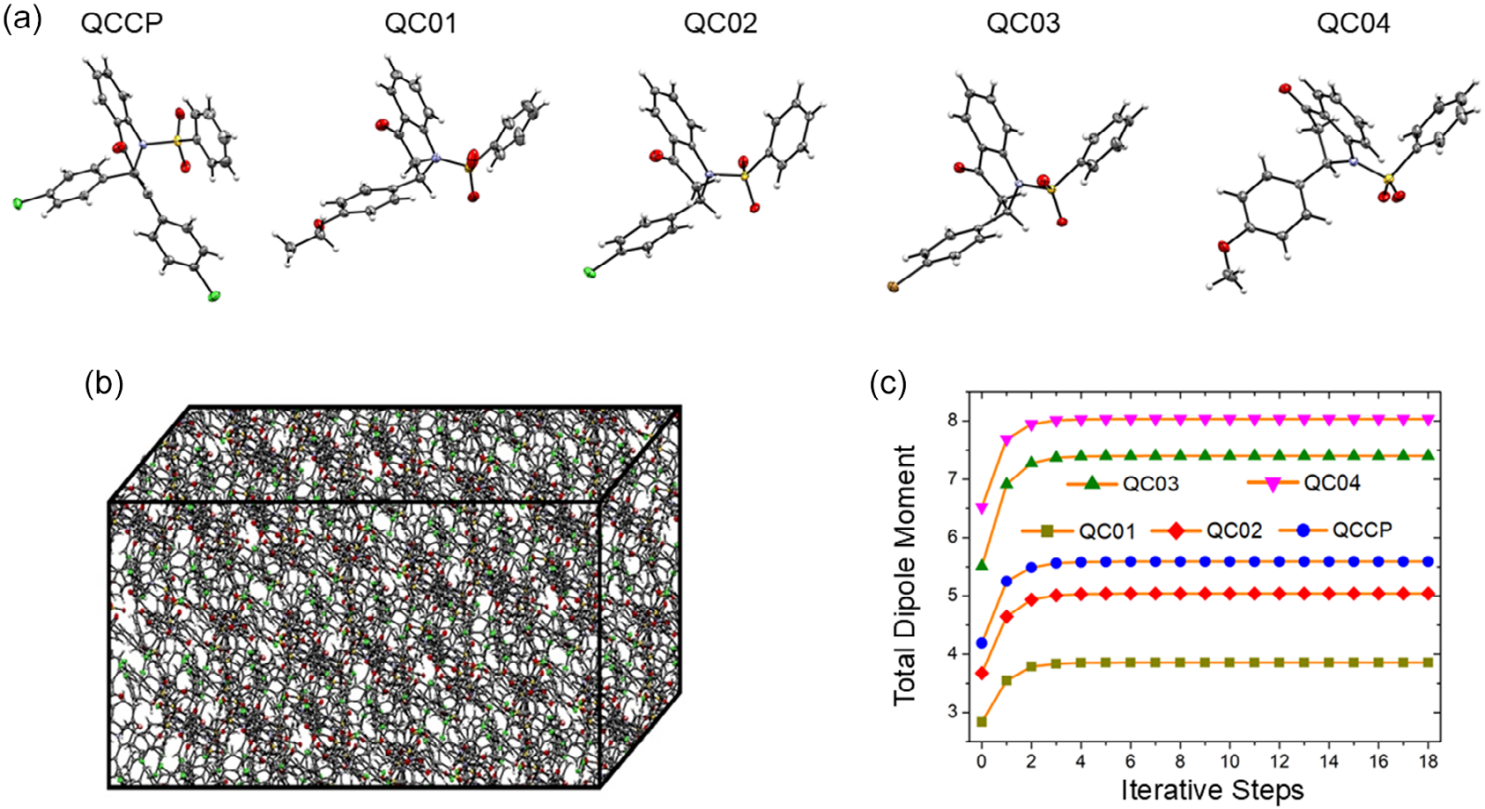
Overview of the computational workflow. (a) ORTEP representations of the asymmetric units (50% probability ellipsoids) for QCCP and QC01–QC04. (b) 17 × 17 × 17 supercell used to reproduce the long‐range crystal electrostatic field in ICE. (c) Convergence of the total molecular dipole moment along ICE iterations; step 0 corresponds to the isolated molecule and the final step to the self‐consistent embedded state.

Figure 1 brings together the main parts of the computational workflow. Figure 1a shows ORTEP drawings for the five quinolinone chalcone structures (QCCP, QC01, QC02, QC03, and QC04) with 50% probability ellipsoids that make their small conformational differences easy to see. Figure 1b displays the 17 × 17 × 17 supercell, which recreates the long‐range electrostatic field acting inside the crystal. The iterative procedure was continued until the variation in the total dipole moment between consecutive steps was less than the tolerance limit of 10^−2^ Debye. While periodic boundary condition calculations offer a full description of the solid state, the ICE method provides a cost‐effective alternative that efficiently captures the essential polarization effects required for NLO property prediction. Figure 1c tracks the dipole moment over 18 self‐consistent cycles, beginning with an isolated molecule at step 0 and ending with the fully embedded form at step 18. After convergence the dipole moments settle at 3.85 D for QC01, 5.04 D for QC02, 7.40 D for QC03, 8.03 D for QC04 and 5.59 D for QCCP, clearly illustrating the strong polarization produced by the crystal lattice.

Figure 1 illustrates that the ICE approach provides a description of the crystal‐field‐induced polarization effects. Such effects are crucial for prediction of NLO properties. In ICE, the crystalline environment is approximated by fixed point charges, so the model captures mainly the electrostatic crystal field but does not include short‐range exchange–repulsion, many‐body environment polarization/induction, dispersion, or explicit intermolecular charge–transfer/excitonic coupling.

### 2.4 Optical Parameters

The total dipole moment was determined according to the expression:

(4) $$\mu =\sqrt{\mu_{x}^{2}+\mu_{y}^{2}+\mu_{z}^{2}}$$

The other linear parameters at electric field frequency (*ω*), such as the dynamic average linear polarizability (⟨α(−ω;ω)⟩) and the linear refractive index (n(ω)), were calculated using the following expressions



(5)

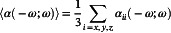

(6) $$n{\left(\omega \right)}^{2}=1+\frac{N}{\varepsilon_{o}V}\left\langle \alpha \left(-\omega ;\omega \right)\right\rangle$$

where N is the number of molecules in the unit cell and V is the unit cell volume, and $\varepsilon_{o}$ is the vacuum permittivity. According Kongsted, Osted, Mikkelsen and Christiansen [59], the average second hyperpolarizability can be calculated by,

(7) $$\left\langle \gamma \right\rangle =\frac{1}{15}\sum_{ij=x,y,z}\left(\gamma_{\mathrm{iijj}}+\gamma_{\mathrm{ijij}}+\gamma_{\mathrm{ijji}}\right)$$

However, when optical dispersion is absent, Kleimann symmetry can be used. This allows for the calculation of the static and dynamic average second hyperpolarizabilities via the expressions below

(8) $$\left\langle \gamma \left(0;0;0;0\right)\right\rangle =\frac{1}{5}\sum_{i,j=x,y,z}\left(\gamma_{iijj}\left(0;0;0;0\right)\right)$$

(9) $$\left\langle \gamma \left(-\omega ;\omega ;0;0\right)\right\rangle =\frac{1}{5}\sum_{i,j=x,y,z}\left(\gamma_{iijj}\left(-\omega ;\omega ;0;0\right)\right)$$

(10) $$\left\langle \gamma \left(-2\omega ;\omega ,\omega ,0\right)\right\rangle =\frac{1}{5}\sum_{i,j=x,y,z}\left(\gamma_{iijj}\left(-2\omega ;\omega ,\omega ,0\right)\right)$$

The third‐order nonlinear susceptibility $\left\langle \chi^{3}(-\omega ;\omega ;\omega ;-\omega )\right\rangle$ is the macroscopic parameter related with the second hyperpolarizability, particularly here the calculation of this parameter involves the Lorentz local field correction factor given by,

(11) $$f=\frac{\left(n^{2}+2\right)}{3}$$

and the average second hyperpolarizability IDRI (intensity‐dependent refractive index) that is defined by the approximation,

(12) ⟨γ(−ω;ω,ω,−ω)⟩≅2⟨γ(−ω;ω,0,0)⟩−⟨γ(0;0,0,0)⟩

Therefore, the dynamic third‐order nonlinear susceptibility is given by,

(13) $$\chi^{\left(3\right)}\left(-\omega ;\omega ,\omega ,-\omega \right)=\frac{f^{4}N}{\in_{o}V}\left\langle \gamma \left(-\omega ;\omega ,\omega ,-\omega \right)\right\rangle$$

Using the Gaussian 16 software package [60], all calculations were computed employing the DFT/CAM‐B3LYP/6‐311++G(d, p) method.

## 3 Results and Discussions

### 3.1 Solid State Description

**SCHEME 2:**
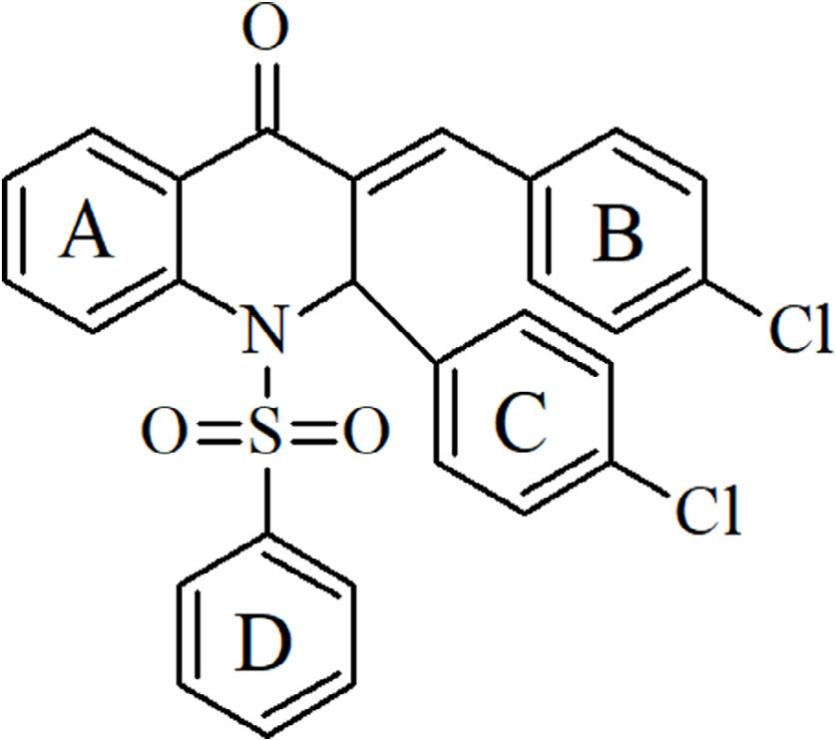
QCCP structural formula.

The first step in our analysis is to provide a detailed description of the molecular and crystallographic structure of QCCP, which serves as the foundation for understanding its nonlinear optical response. QCCP is a quinolinone‐chalcone hybrid originally synthetized by d’Oliveira and coworkers [37]. QCCP falls into the category of molecules described as quinolinone‐chalcone hybrids, which frequently have substituents on rings B and C. In the case of QCCP, two chlorine substituents are located specifically at the para positions of rings B and C (see Scheme 2). The presence of a stereogenic center at carbon C10 leads to optical isomerism. Given that the crystal is centrosymmetric, it crystallizes as a racemate. Therefore, any interactions examined are observed in both its enantiomeric forms.

The QCCP crystal belongs to orthorhombic centrosymmetric space group *Pbca*, with eight molecules in unit cell and a single molecule in asymmetric unit, with *a* = 20.185(2), *b* = 11.2428 (12) Å, *c* = 21.591 (2) Å, *α* = 90º, *β* = 90º, *γ* = 90º, *Z* = 8, and unit cell volume, *V* (Å^3^) = 4899.8 (9). More experimental information about the crystal and refinement are provided in Supplementary Information, Table S1 (Supplementary Information, SI). The conformation of QCCP molecule in the crystal can be described by torsion angles presented in Table S2 (SI).

**FIGURE 2:**
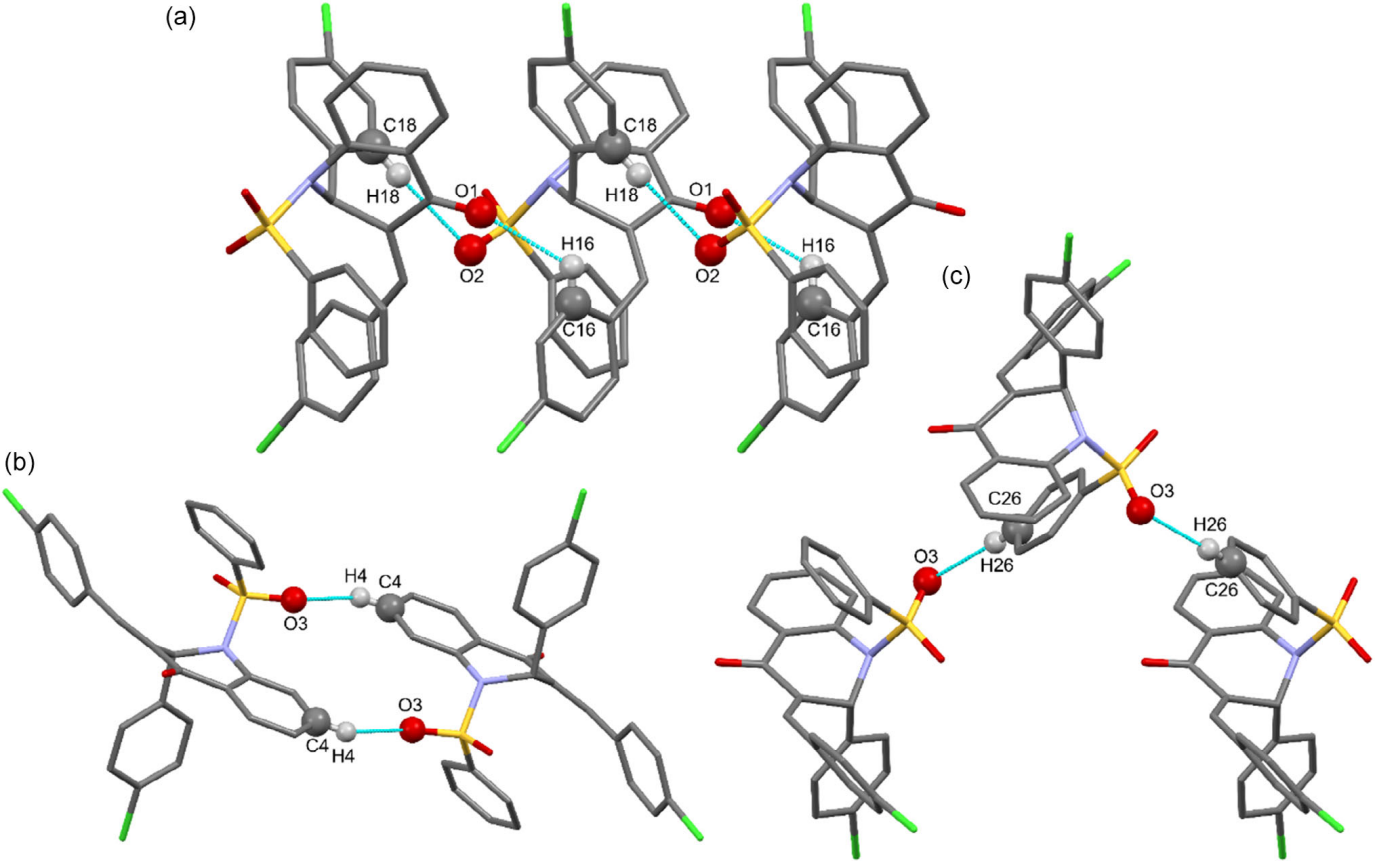
Molecular planes generated by the (a) C16–H16···O1 and C18–H18···O2 contacts; (b) are stacked through dimers linked by the C4–H4···O3 interaction; and (c) are further connected by the C26–H26···O3 contact.

The strongest intermolecular forces that stabilize the crystal are C–H···O interactions. First, the C16–H16···O1 and C18–H18···O2 contacts align related molecules along the [010] direction (Figure 2a; Table S3, SI). Next, each molecule forms a dimer stacked along [001], mediated by the C4–H4···O3 interaction (Figure 2b). Finally, the C26–H26···O3 contacts propagate molecules along [100] via a 2‐fold screw axis; together with the previous interactions, they generate layers that lie in the [001] plane (Figure 2c).

**FIGURE 3:**
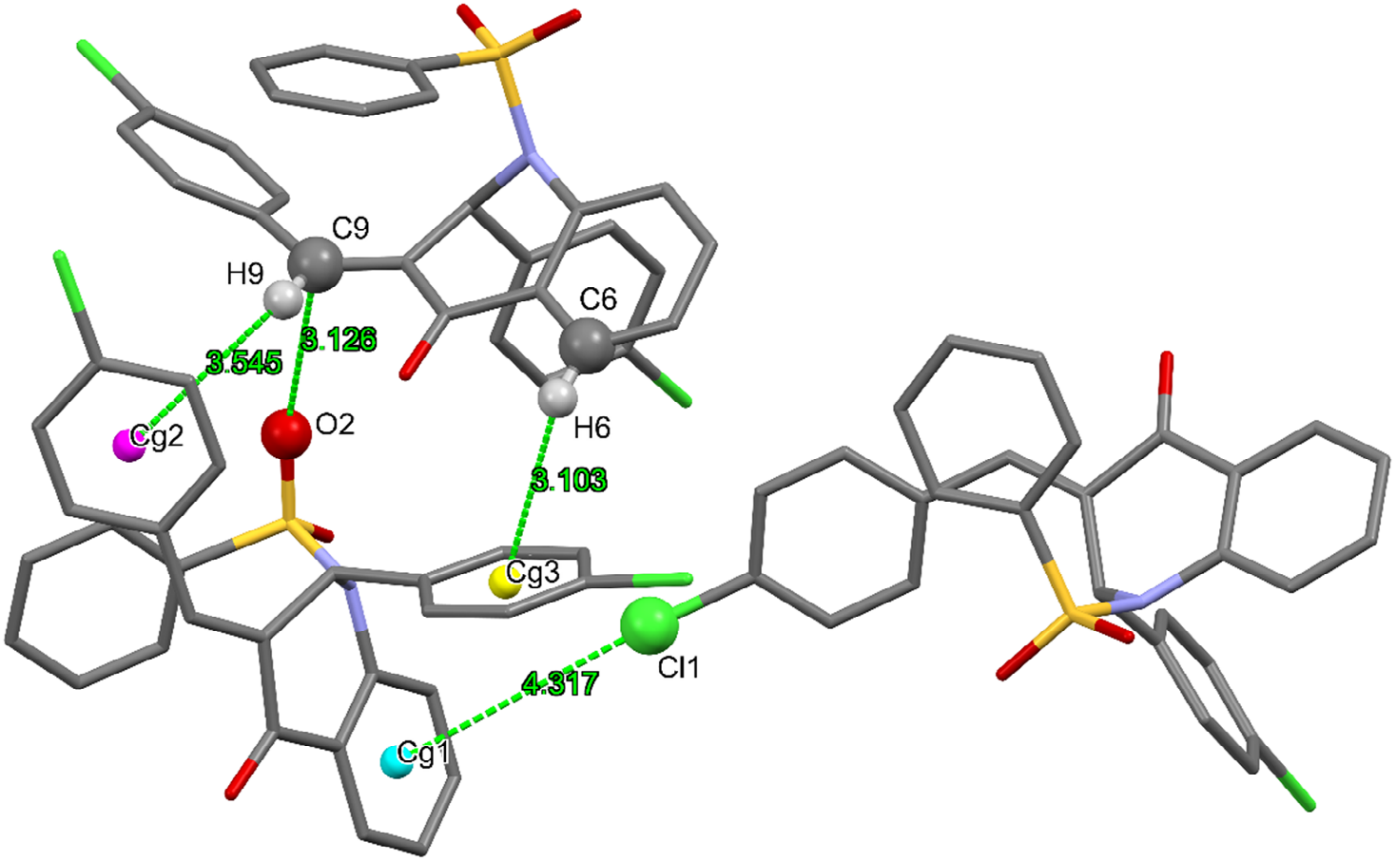
Weak interactions between *π* systems and halogen, oxygen, or hydrogen.

Weaker interactions are also present in this crystal, like the ones involving *π* systems shown in Figure 3. C6–H6···Cg3 and C9–H9···Cg2 interactions contributes to stabilization of contacts described previously in Figure 2a. The distance between atoms also suggests a O···*π* interactions between O2 and C9, reinforcing contacts in this direction. On the other hand, Cl1···Cg1 interaction assists in the layer stacking of the crystal. Some geometric parameters of these interactions are listed in Table S4 (SI).

**FIGURE 4:**
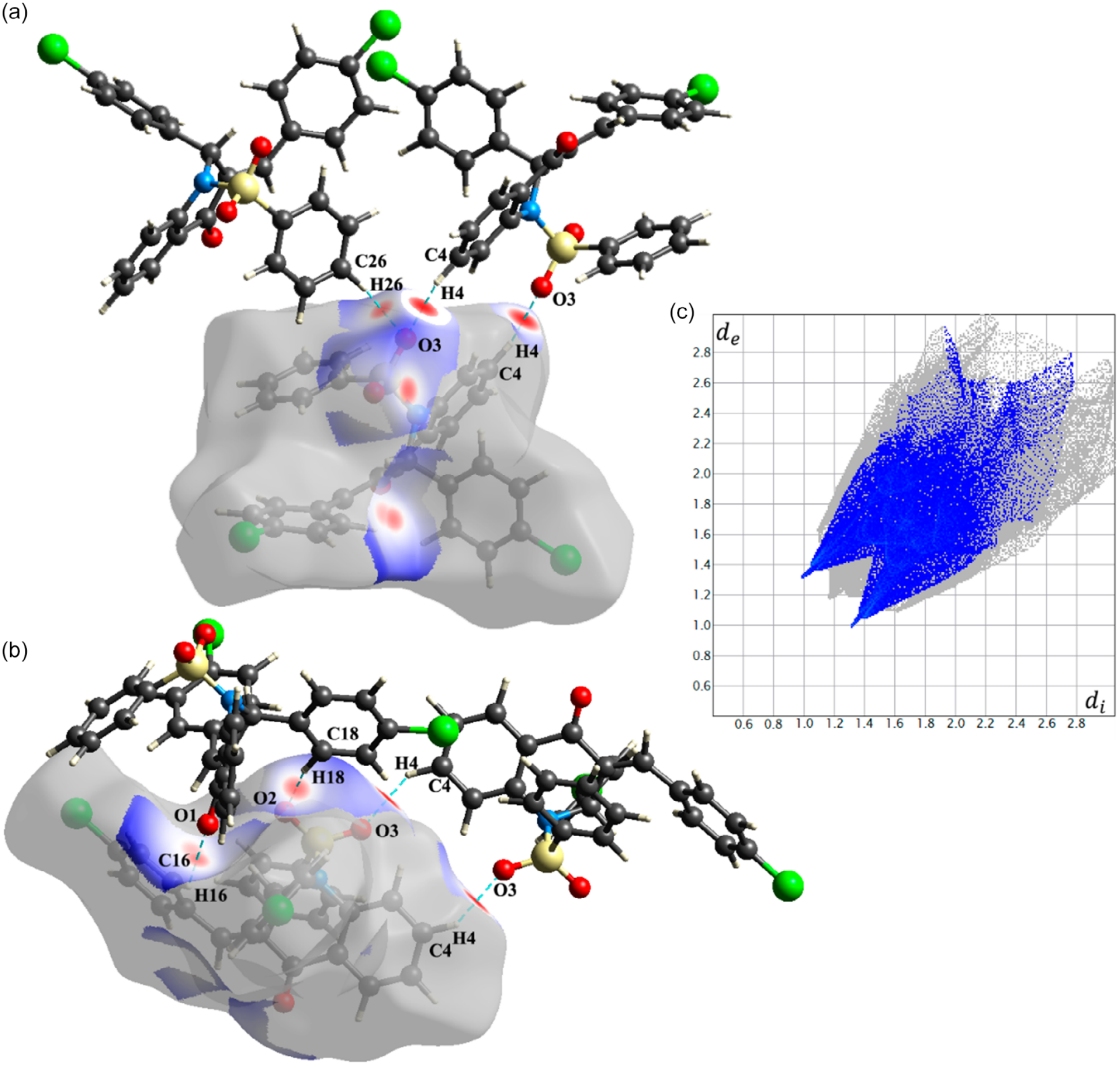
Hirshfeld surface analysis of QCCP highlighting dominant intermolecular contacts. (a,b) *d*_norm_ surfaces showing the main C–H···O interactions as red regions (stronger/shorter contacts). (c) 2D fingerprint plot summarizing (d_i_, d_e_) contact distributions, emphasizing O···H/H···O contributions to crystal packing.

The analysis of Hirshfeld Surface reinforces the presence of described interactions. Figure 4 shows the *d*_norm_ property of the surface, where intermolecular interactions are presented as red spots between the atoms involved. The size and intensity of color is proportional to strength of the interaction. C26‐H26···O3 and C4‐H4···O3 bifurcated interactions is presented in Figure 4a, while Figure 4b shows interactions C16‐H16···O1 and C18‐H18···O2. The fingerprint plot in Figure 4c, a diagram of frequencies of d_e_ and d_i_, is highlighting the H···O and O···H interactions.

In addition, these intermolecular contacts contribute to the local electrostatic environment and local field factors, which may influence the third‐order optical response; however, in a centrosymmetric *Pbca* space group, they do not result in a net macroscopic polar alignment of molecular dipoles.

### 3.2 Molecular Modeling Analysis

Understanding the chemical reactivity profile of molecules involves considering thermodynamic principles and attributes like polarizability and rigidity. Various parameters help clarify how molecules interact in chemical reactions and their ability to donate or accept electrons. Among these are global descriptors, which serve as indicators for characterizing a substance's chemical properties on a broader molecular scale. These descriptors are derived from the energies of the frontier molecular orbitals, namely the highest occupied molecular orbital (HOMO) and the lowest unoccupied molecular orbital (LUMO). Table 1 shows the ionization energy (IE), the electron affinity energy (EA), the global hardness (*η*), the chemical potential (μ_p_), the global softness (*σ*) and the overall electrophilicity (*ϖ*). The *ϖ*‐value is connected to the charge transfer process, and the propensity of a molecule to donate (*ϖ*−) or accept (*ϖ*+) an electron can be established from these descriptors.

**TABLE 1:** Global Reactivity descriptors (eV) for QCCP isolated and embedded molecules.

Descriptors	Isolated	Embedded
*ε*_HOMO_	−8.31	−8.67
*ε*_LUMO_	−1.65	−2.03
Ionization energy *I*_*E*_= −*ε*_*HOMO*_	8.31	8.67
Electron affinity *A*_*E*_ = −*ε*_*LUMO*_	1.65	2.03
Global hardness *η* = (*I*_*E*_ − *A*_*E*_)/2	3.33	3.32
Chemical potential *μ*_p _= ((*I*_*E*_ + *A*_*E*_)/2)	4.98	5.35
Global softness *σ* = 1/*η*	0.30	0.30
Global electrophilicity *ϖ *= *μ* ^2^/2*η*	3.73	4.31
Electron donating capability $\varpi^{-}=\frac{{\left(3I_{E}+A_{E}\right)}^{2}}{16\left(I_{E}-A_{E}\right)}$	6.63	7.40
Electron accepting capability $\varpi^{+}=\frac{{\left(I_{E}+3A_{E}\right)}^{2}}{16\left(I_{E}-A_{E}\right)}$	1.65	2.05

As can be noted from Table 1 the global reactivity descriptors are benefit by the crystalline environment polarization effect, all parameters increase in magnitude, except the global softness that remains stable and the global hardness that present a smaller decreasing. The QCCP isolated and embedded molecules can be classified as chemically hard with a greater kinetic stability and electron donating capacity. The capability of donating and accepting electrons present an increasing of 11.6% and 24% in crystalline phase, as compared with the results for isolated molecule.

Based on frontier molecular orbital (HOMO/LUMO) analysis and molecular electrostatic potential (MEP) maps, the electronic structure of QCCP was examined in both isolated and embedded states, with the images of the HOMO/LUMO orbitals (Figure S5) and MEP (Figure S6) provided in the Supplementary Information**.** The analysis of the frontier molecular orbitals (Figure S5) reveals a distinct charge transfer character. The HOMO is predominantly localized on the donor quinolinone moiety, while the LUMO is shifted towards the acceptor sulfonyl and chalcone bridge. This spatial redistribution of electron density upon excitation facilitates the intramolecular charge transfer process, which is a key driver for the observed enhancement in NLO response.

Orbital analysis showed the distribution of HOMO (primarily on rings B, C, nitrogen, and chlorines) and LUMO (spanning rings A, B, C, D, chlorines, and sulfur). Embedding resulted in a small decrease in the energy gap (Δ*E*), from 6.66 to 6.64 eV, attributed to crystalline environment polarization. MEP maps revealed the distribution of electrostatic potential, with areas near oxygen atoms being most negative, hydrogen atoms most positive, and chlorine atoms displaying intermediate potential. Our results regarding charge transfer and orbital stabilization align with recent findings in material design. Ali et al. [61]. recently highlighted, through FMO and NBO analyses, that minimizing the energy gap and maximizing electrostatic complementarity are crucial for the stability and reactivity of ionic systems, a trend we also observe in the polarized environment of the QCCP crystal.

### 3.3 Charge Transfer in the Embedded QCCP Molecule

Table S5 (SI) shows the charge of selected groups of the isolated and embedded molecules of QCCP. The magnitude of the charge of the group 1 and group 2 increases and of the group 3 and group 4 decreases, due the crystalline environment polarization effects, keeping the same signal. The charge of the subgroups 4a (SO_2_) increases in magnitude, and the charge signal change from positive to negative due to the crystalline environment polarization effect, which also increases the magnitude of the negative charge of the subgroup 1a (O_1_).

**FIGURE 5:**
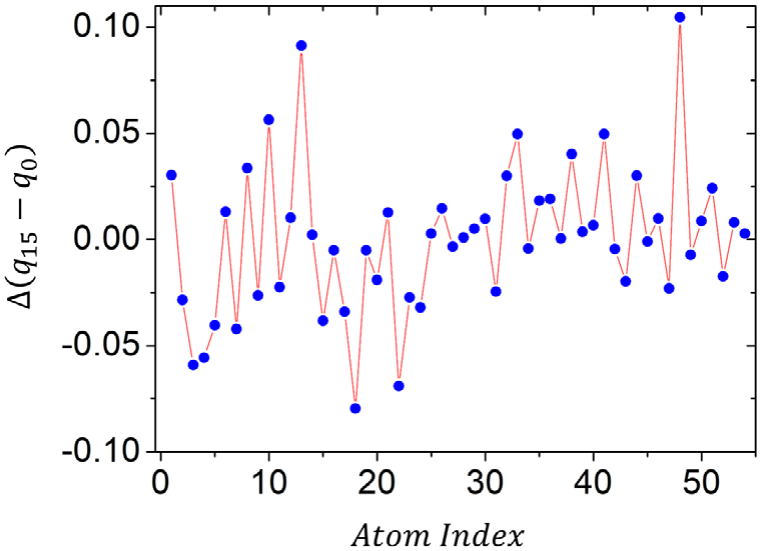
The *x*‐axis represents the index of each atom in the molecular structure, while the *y*‐axis shows the difference in partial charge (Δ*q* = q_15_ – q_0_) using CHELPG charges, where step 0 is the isolated molecule and step 15 is the converged ICE embedded state.

To further the analysis, atomic charges of the QCCP molecule were evaluated individually. The overall average charge variation was approximately 0.0000, with a standard deviation of 0.035, indicating balanced charge redistribution with localized effects. The largest increase occurred for a hydrogen atom (index 48), which changed from + 0.0818 to + 0.1865, suggesting stronger polarization in its local environment. As shown in Figure 5, the partial charge variation (Δ*q*) for each atom of the QCCP molecule between iteration step 0 and step 15 highlights the distribution of these effects.

The *x*‐axis corresponds to the atom index listed in Table S6 (index–element mapping and per‐atom charges). The functional‐group charge balance associated with the same redistribution is summarized in Table S5 (selected fragments; isolated vs embedded). Positive Δq indicates the site becomes more positive (electron depletion), whereas negative Δq indicates electron accumulation, according to the CHELPG sign convention. Positive values indicate electron depletion (the site becomes more positive), whereas negative values indicate electron accumulation. The plot reveals regions of significant polarization induced by the crystalline environment. Particularly, carbon atom 13 exhibits charge inversion, shifting from –0.041 to + 0.050, reflecting a clear reversal in the local electrostatic potential. The complete list of atomic charges for QCCP is provided in Table S6 (SI). These data reinforce that the crystalline environment substantially alters the electronic distribution within the molecule, supporting the discussion in Table S5 (SI) and highlighting the effectiveness of the embedding approach in capturing these subtle effects.

### 3.4 NLO Properties

Table 2 shows the DFT/CAM‐B3LYP/6‐311++G(d, p) results for the linear refractive index (n(*ω*)), the average linear polarizability (*α*(‐*ω*;*ω*)), the second average hyperpolarizabilities *γ*(‐*ω*;*ω*, 0,0), *γ*(‐2*ω*;*ω*,*ω*, 0) and *γ*(‐*ω*;*ω*,*ω*,‐*ω*) and for the third‐order nonlinear susceptibility (*χ*3(‐*ω*;*ω*,*ω*,‐*ω*)) for embedded molecules of the QCCP compound in the static and dynamic regime, also is shown in Table 2 the electric parameter values for the other four compounds QCCP, QC01, QC02, QC03, and QC04 at 532 nm. As can be seen the linear parameters of QCCP are practically insensitive to the increase of the electric field frequencies, the *α*‐value and *n*‐value at *λ*=532 nm is only 8% and 3.2% greater than the respective static value. The value of *γ*(‐*ω*; *ω*, 0,0) present a perceptual increase of 15% and 96% at the electric field wavelength of 1064 and 532 nm in comparison with static case. The γ‐value at 532 nm $81.48\times 10^{-36}esu$ is a significant value, greater than observed for some chalcones‐derivative. Likewise, the IDRI second hyperpolarizability shows a growth of 30% and 192% compared to the static case, reaching a value of $121.39\times 10^{-36}esu$ at 532 nm.

**TABLE 2:** Static and dynamic optical parameters for the embedded QCCP molecule (static, 1064 and 532 nm) and for QC01–QC04 at 532 nm. n(*ω*) is dimensionless. *α*(−*ω*; *ω*) is given in 10^−24^ esu. The average second hyperpolarizabilities *γ*(−*ω*; *ω*, 0,0), *γ*(−2*ω*; *ω*,*ω*, 0) and *γ*(−*ω*; *ω*,*ω*,−*ω*) are given in 10^−36^ esu. The third‐order nonlinear susceptibility *χ* ^(3)^(−*ω*; *ω*,*ω*,−*ω*) is given in ($10^{-22}{\left(m/V\right)}^{2}$).

	QCCP			QC01	QC02	QC03	QC04
Electric parameters	Static	*λ *= 1064nm	*λ *= 532nm	*λ *= 532nm	*λ *= 532nm	*λ *= 532nm	*λ *= 532nm
n(*ω*)	1.58	1.59	1.63	1.65	1.68	1.72	1.72
*α*(‐*ω*;*ω*)	48.45	49.28	52.29	43.19	41.36	41.27	42.63
*γ*(‐*ω*;*ω*, 0,0)	41.57	47.84	81.48	47.51	45.29	46.17	45.02
*γ*(‐2*ω*;*ω*,*ω*, 0)	41.57	66.90	219.42	1410.26	370.63	140.00	142.35
*γ*(‐*ω*;*ω*,*ω*,‐*ω*)	41.57	54.11	121.39	61.51	58.80	58.49	57.17
χ^3^(‐*ω*;*ω*,*ω*,‐*ω*)	47.42	63.86	162.52	107.59	119.13	142.95	132.28

Regarding linear properties, QCCP, with its more extensive *π*‐system, shows the highest molecular polarizability (*α* ≈ 52 × 10^−24^ esu) as compared with the values for others compounds at 532 nm, shown in Table 2. In contrast, the halogenated derivatives QC03 and QC04 present the highest refractive index (*n* ≈ 1.72), a reflection of their more compact packing and the high atomic number of the halogens. For the second‐order nonlinear properties, QCCP leads in the hyperpolarizabilities *γ*(‐*ω*;*ω*, 0,0) and *γ*(‐*ω*;*ω*,*ω*,‐*ω*), confirming the decisive role of extended conjugation. However, QC01 shows an extraordinary peak in *γ*(‐2*ω*;*ω*,*ω*, 0) (≈ 1.4 × 10^3^ × 10^−36^ esu), attributed to a two‐photon resonance induced by its electron‐donating ethoxy group.

The QCCP third order nonlinear susceptibility value at *ω *= 0.043 a.u. (λ=532 nm) is $162.52\times 10^{-22}{\left(m/V\right)}^{2}$. This is a significant value, mainly when compared with the experimental values obtained for chalcone derivatives (see Table 3), indicating that the QCCP crystal has interesting NLO properties and has the potential to be studied as an NLO material. In macroscopic terms, while QCCP reaches the highest *χ* ^3^ value, it is noteworthy that QC03 and QC04 come close to this performance. Their high performance is due to a combination of high refractive index and greater molecular density, which are factors that amplify the local field. This study highlights that the nonlinear optical response results from a synergy between extending conjugation to maximize *γ*, adjusting substituents to explore specific resonances, and engineering the crystal packing to enhance local field effects.

**TABLE 3:** Third‐order nonlinear optical susceptibility for QCCP crystal compared with the dynamic experimental (Exp) and *theoretical* (*Theo*) *results* (λ=532nm) for some organic nonlinear crystals.

	λ(nm)	$\chi^{\left(3\right)}\left(10^{-22}{\left(\frac{m}{V}\right)}^{2}\right)$
QCCP (present work)	532	162.52 – Theo
QC01 (present work)	532	107.59 – Theo
QC02 (present work)	532	119.13 – Theo
QC03 (present work)	532	142.95 – Theo
QC04 (present work)	532	132.28 – Theo
4‐[(1E)‐3‐(3,4‐dimethoxyphenyl)‐3‐oxoprop‐1‐en‐1‐yl]phenyl 4‐methylbenzene‐1‐sulfonate [62]	532	433– Exp
(2E)‐3‐(3‐methylphenyl)‐1‐(4‐nitrophenyl)prop‐2‐en‐1‐one (3MPNP) [63]	532	277.1 – Exp
1‐(5‐chlorothiophen‐2‐yl)‐3‐(2,3‐dimethoxyphenyl)prop‐2‐en‐1‐one (CTDMP) [63, 64]	532	23.83 – Exp
Zinc(II) complex Zn [65]	532	21.38 – Exp
N‐(2‐hydroxyphenyl)‐3‐hydroxy‐4‐iminocyclohexa‐2,5‐dien‐1‐one [65]	532	15.16 – Exp
Nickel(II) complex Ni [65]	532	14.39 – Exp
Copper(II) complex Cu [65]	532	6.07 – Exp
(2E)‐3[4(methylsulfanyl)phenyl] ‐1‐(4‐nitrophenyl)prop‐2‐en‐1‐ one (4N4MSP) [63, 64]	800	2.37 – Exp
(2E)‐1‐(4‐bromophenyl)‐3‐[4(methylsulfanyl)phenyl]prop‐2‐en‐1‐one (4Br4MSP) [63, 66]	800	2.30 – Exp
(2E)‐1‐(3‐bromophenyl)‐3‐[4(methylsulfanyl)phenyl]prop‐2‐en‐1‐one (3Br4MSP) [63, 64]	800	1.99 – Exp

As shown in Table 3, some well‐known chalcone derivatives, such as 3MPNP, present higher *χ* ^(3)^‐values ($277.1\times 10^{-22}{\left(m/V\right)}^{2}$), while others, including CTDMP ($23.83\times 10^{-22}{\left(m/V\right)}^{2}$) and 4N4MSP ($2.37\times 10^{-22}{\left(m/V\right)}^{2}$), exhibit considerably lower responses. Notably, many of the reported values are measured at 800 nm (*ω *= 0.057 a.u.), where the non‐resonant contribution tends to dominate. Nonlinear effects at this wavelength are typically weaker due to the lower absorption cross‐section and reduced resonance contribution at longer wavelengths. Therefore, these comparisons should be considered qualitative, as they are influenced by experimental conditions such as wavelength‐dependent absorption and resonance effects.

Despite the frequency dependence of *χ* ^(3)^(‐*ω*;*ω*,*ω*,‐*ω*) the value reported for QCCP at 532 nm is comparable or superior to the majority of experimental data for chalcone‐based systems, suggesting a robust nonlinear response in the visible region. Moreover, the magnitude of *χ* ^(3)^ in QCCP indicates that its electronic structure and crystalline packing favor significant delocalization and polarizability, which are essential for enhanced third‐order NLO activity. These findings position the QCCP crystal as a promising candidate for nonlinear optical applications, particularly in integrated optics and photonics operating within the visible spectrum. Future experimental studies, such as Z‐scan or third‐harmonic generation measurements, are encouraged to validate the theoretical predictions and explore the crystal's behavior under high‐intensity fields.

Regarding the optical response, the tuning of NLO properties via structural modification is a key strategy. Ibrahim et al. [67] showed that subtle structural changes, such as H‐migration in planar heterocycles, can significantly modulate aromaticity and hyperpolarizability. Similarly, our study confirms that substituting the para‐position in the chalcone moiety effectively tunes the response. Moreover, Ullah et al. [68] demonstrated that enhancing charge transfer pathways is essential for high‐performance NLO materials in sensing applications. The high value obtained for QCCP suggests efficient intramolecular charge transfer, making it a competitive candidate for similar advanced optoelectronic devices.

## 4 Conclusion

The crystallographic structure of QCCP was determined by single‐crystal X‐ray diffraction, revealing that the compound crystallizes in the orthorhombic system with space group *Pbca*. The unit cell parameters are: *a* = 20.185(2) Å, *b* = 11.2428(12) Å, *c* = 21.591(2) Å, with a unit cell volume of 4899.8(9) Å^3^. The crystal structure is characterized by a layered arrangement of molecules, predominantly stabilized by *π*–*π* stacking and C–H···O interactions. This spatial organization results in preferential alignment of molecular dipoles along specific crystallographic axes, which is a crucial factor in enhancing the macroscopic polarizability and, consequently, the nonlinear optical response of the crystal. The molecular conformation is preserved within the crystal, and no significant disorder or solvent inclusion was observed in the asymmetric unit. These structural characteristics support the charge transfer efficiency and the strong dipolar behavior observed in the embedded system.

To simulate the effect of the crystalline environment on the electronic structure, the ICE method was employed, which revealed significant polarization effects that impacted atomic charge distribution and increased the total dipole moment from 3.86 D for QC01, 5.04 D for QC02, 7.40 D for QC03, 8.03 D for QC04 and 5.59 D for QCCP. This ICE‐driven charge redistribution sharpens donor–acceptor separation along the *π*‐conjugated framework, a prerequisite for strong nonlinear behavior. Using the embedded charge maps, we then evaluated third‐order optical parameters under both static and 532 nm fields, finding that every embedded model outperforms its isolated analog.

This redistribution of charge also influenced the electronic structure, enhancing the electron‐donating and accepting capabilities of the molecule. The quinolinone–chalcone family as a whole shows remarkable third‐order nonlinear performance at 532 nm. Besides QCCP, whose third‐order nonlinear susceptibility value is $162.52\times 10^{-22}{\left(m/V\right)}^{2}$, at 532 nm, the dihydroquinolinone derivatives also respond strongly with high *χ* ^3^: QC03 ($142.95\times 10^{-22}{\left(m/V\right)}^{2}$), QC04 ($132.28\times 10^{-22}{\left(m/V\right)}^{2}$), QC02 ($119.13\times 10^{-22}{\left(m/V\right)}^{2}$), and QC01 ($107.59\times 10^{-22}{\left(m/V\right)}^{2}$). Every value surpasses most chalcone‐based crystals reported so far, confirming that structural variations around the quinolinone core can deliver high *χ* ^3^‐value, without sacrificing chemical robustness or crystallographic stability. The ICE approach proved essential for capturing the local‐field amplification produced by dense packing, underscoring its value for predicting solid‐state optical behavior. Taken together, these results spotlight quinolinone‐chalcone derivatives as a versatile platform for visible‐region photonic devices and justify further experimental work to translate the computational predictions into practical components.

## Supporting Information

Additional supporting information can be found online in the Supporting Information Section. **Supporting Fig. S1:** ^1^H NMR spectrum (500 MHz, CDCl_3_) of compound QCCP. **Supporting Fig. S2:** ^13^C{^1^H} NMR spectrum (126 MHz, CDCl_3_) of compound QCCP. **Supporting Fig. S3:** High‐resolution mass spectrum of compound QCCP. **Supporting Fig. S4:** Infrared spectrum of compound QCCP. **Supporting Fig. S5:** HOMO and LUMO frontiers orbitals and the gap energies for (a) isolated and (b) embedded QCCP molecules. **Supporting Fig. S6:** MEP for both (a) isolated and (b) embedded molecules of QCCP. **Supporting Table S1:** Experimental details of QCCP crystal. **Supporting Table S2:** Main torsion angles from QCCP molecule. **Supporting Table S3:** Geometric parameters of C‐H···O interactions from QCCP. **Supporting Table S4:** Geometric parameters of C‐H··· π, halogen··· π and O··· π interactions from QCCP. **Supporting Table S5:** Charges (e) of selected groups of isolated and embedded molecules of QCCP. **Supporting Table S6:** Atomic charges of the QCCP molecule at steps 0 and 15 of the iterative embedding procedure, along with the corresponding charge differences (Δq = q_15_ – q_0_). Atom indices correspond to the molecular structure presented in the main text. These data support the charge redistribution analysis and the polarization effects discussed in Section 4.3 and Table 6.

## Author Contributions

All authors contributed to the study conception and design. The experiments were carried out by **Vitor S. Duarte**, **Giulio D. C. D’Oliveira**, **Jean M. F. Custodio**, **Caridad N. Pérez**, and **Hamilton B. Napolitano**. The theoretical calculations were performed by **Clodoaldo Valverde**, **Nathália M. Pires**, **Antônio N. Borges**, **Daphne C. Fernandes**, and **Francisco A. P. Osório**. The first draft of the manuscript was written by **Clodoaldo Valverde**, **Nathália M. Pires**, **Antônio N. Borges**, **Daphne C. Fernandes**, **Vitor S. Duarte**, **Giulio D. C. D’Oliveira**, **Jean M. F. Custodio**, **Caridad N. Pérez**, **Francisco A. P. Osório**, **Hamilton B. Napolitano**. and all authors commented on previous versions of the manuscript. All authors read and approved the final manuscript.

## Conflicts of Interest

The authors declare no conflicts of interest.

## Supporting information

Supplementary Material

## Data Availability

The data that support the findings of this study are available in the supplementary material of this article.
