# Supplementary material for: Theoretical Study of Electrostatic Embedding and Properties of a Novel Quinolinone‐Chalcone Crystal and a Comparative Analysis with Dihydroquinolinone Analogs
Source: ChemistryOpen. 2026 Feb 8;15(2):e202500527. doi: 10.1002/open.202500527 (PMC12883574; doi:10.1002/open.202500527)
Supplement: Supplementary file 1 — Supplementary Material [file OPEN-15-e202500527-s001.pdf]

Theoretical Study of Electrostatic Embedding and Nonlinear Optical Properties of a Quinolinone–Chalcone Crystal

Clodoaldo Valverde[1,2], Nathália M. Pires[2], Antônio N. Borges[3], Daphne C. Fernandes[2], Vitor S. Duarte[1], Giulio D. C. D'Oliveira[4], Jean M. F. Custodio[5], Caridad N. Pérez[4], Francisco A. P. Osório[6], Hamilton B. Napolitano[1,7]

[1] Universidade Estadual de Goiás, 75001-970, Anápolis, GO, Brasil
[2].Universidade Paulista – UNIP, 74845-090, Goiânia, GO, Brasil
[3] Pontifícia Universidade Católica de Goiás, 74605-100, Goiânia, GO, Brasil.
[4] Instituto de Química, Universidade Federal de Goiás, Goiânia, GO, Brasil.
[5] Departament of Chemistry and Biochemistry,University of Notre Dame, 46556, Notre Dame, IN, USA
[6] Instituto de Física, Universidade Federal de Goiás, 74.690-900, Goiânia, GO, Brasil.
[7] Universidade Evangélica de Goiás, 75083-515, Anápolis, GO, Brasil.

Abstract

In this work we study the linear and nonlinear optical properties of a quinolinone-chalcone derivative 4(1H)-quinolinone-(E)-4-chlorobenzylidene-4-chlorophenyl-phenylsulfonyl with formula $C_{28}H_{19}Cl_2NO_3S$. Theoretical calculations of the electrical properties of the 4(1H)-quinolinone-(E)-4-chlorobenzylidene-4-chlorophenyl-phenylsulfonyl crystal were carried out at Density Functional Theory (DFT) level with the functional CAM - $B3LYP$ and the basis set 6-311++$G(d, p)$, both, in the static and dynamic regime. To simulate the crystalline environment, we applied an electrostatic iterative embedding approach, known as the Iterative Charge Embedding method, which revealed the significant redistribution of electronic density due to the crystalline polarization effects. Electronic descriptors such as HOMO and LUMO energies, global reactivity parameters, and molecular electrostatic potentials were evaluated. The nonlinear optical response, particularly the third-order macroscopic susceptibility $\chi^{(3)}$, was significantly enhanced in the embedded system, reaching a value of 162.52×10^{-22} $(m/V)^2$ at 532 nm. This result is comparable or superior to the experimental values of well-known chalcone derivatives, positioning the 4(1H)-quinolinone-(E)-4-chlorobenzylidene-4-chlorophenyl-phenylsulfonyl crystal as a promising candidate for nonlinear optical applications.

Supplementary information

Table S1. Experimental details of QCCP crystal.

**Crystal data**	
Chemical formula	$C_{28}H_{19}Cl_2NO_3S$
M_r	520.40
Crystal system, space group	Orthorhombic, *Pbca*
Temperature (K)	120
a, *b*, *c* (Å)	20.185 (2), 11.2428 (12), 21.591 (2)
α , β, γ (°)	90
V ($Å^3$)	4899.8 (9)
Z, Z'	8, 1
Radiation type	Mo *K*α
μ (mm^{-1})	0.38
Crystal size (mm)	0.23 × 0.19 × 0.09
Diffractometer	Bruker *APEX*-II CCD
No. of measured, independent and observed [$I > 2\sigma(I)$] reflections	38529, 2970, 2451
R_{int}	0.056
$(\sin \theta/\lambda)_{max}$ ($Å^{-1}$)	0.526
**Refinement**	
$R[F^2 > 2\sigma(F^2)]$, $wR(F^2)$, S	0.028, 0.066, 1.03
No. of reflections	2970
No. of parameters	393
H-atom treatment	All H-atom parameters refined
$\Delta\rho_{max}$, $\Delta\rho_{min}$ (e $Å^{-3}$)	0.18, −0.30

Table S2. Main torsion angles from QCCP molecule.

Torsion	Sequence of atoms	Angle (º)	Conformation
ω_1	C7—C8—C9—C11	-175.3(2)	antiperiplanar
ω_2	C8—C9—C11—C12	141.0(2)	+ anticlinal
ω_3	C7—C8—C10—C17	-76.7(2)	- synclinal
ω_4	C2—N1—C10—C17	70.4(2)	+ synclinal
ω_5	C8—C10—C17—C18	-28.3(3)	synperiplanar
ω_6	C1—C2—N1—S1	-115.2(2)	- anticlinal
ω_7	S1—N1—C2—C23	-36.3(1)	- synclinal
ω_8	N1—S1—C23—C24	103.8(2)	- anticlinal

Table S3. Geometric parameters of C-H···O interactions from QCCP.

D-H···A	D-H (Å)	H···A (Å)	D···A (Å)	D-H···A (°)	Symmetry code
C16-H16···O1	0.89(2)	2.53(2)	3.117(3)	124(2)	1/2-x,-1/2+y,z
C18-H18···O2	0.91(2)	2.58(2)	3.460(3)	165(2)	1/2-x,1/2+y,z
C4-H4···O3	0.93(2)	2.40(2)	3.225(3)	147(2)	1-x,1-y,1-z
C26-H26···O3	0.97(2)	2.53(2)	3.493(3)	171(2)	1-x,1/2+y,1.5-z

Table S4. Geometric parameters of C-H··· π, halogen··· π and O··· π interactions from QCCP.

X···Cg	Length (Å)	Symmetry code
H6···Cg3	3.103(5)	1/2-x,1/2+y,z
H9···Cg2	3.545(6)	1/2-x,1/2+y,z
O2···C9	3.126(3)	1/2-x,-1/2+y,z
Cl1···Cg1	4.317(6)	1/2-x,1-y,1/2+z

Table S5. Charges (e) of selected groups of isolated and embedded molecules of QCCP.

	Groups	Isolated	Embedded	Ratio Embedded/Isolated
**1**	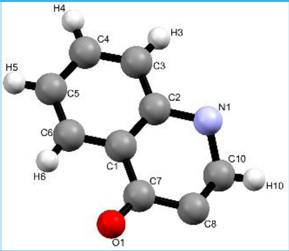	-0.042	-0.113	2.717
**1a**	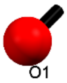	-0.521	-0.576	1.107
**2**	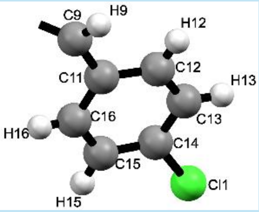	0.115	0.229	1.992
**3**	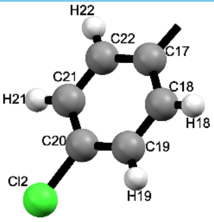	-0.123	-0.119	0.965
**4**	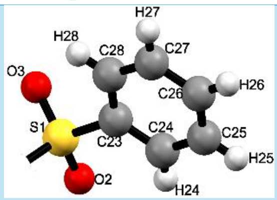	0.050	0.003	0.061
**4a**	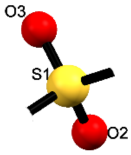	0.0273	-0.042	-1.533

Tabel S6: Atomic charges of the QCCP molecule at steps 0 and 15 of the iterative embedding procedure, along with the corresponding charge differences ($\Delta q = q_{15} - q_0$). Atom indices correspond to the molecular structure presented in the main text. These data support the charge redistribution analysis and the polarization effects discussed in Section 4.3 and Table 6.

Index	Element	Charge_0	Charge_15	Delta_Charge
**1**	S	1.179386	1.209698	0.030312
**2**	Cl	-0.143794	-0.172272	-0.028478
**3**	O	-0.571478	-0.630553	-0.059075
**4**	O	-0.52083	-0.576441	-0.055611
**5**	O	-0.580576	-0.621055	-0.040479
**6**	N	-0.557282	-0.544154	0.013128
**7**	C	0.374329	0.332091	-0.042238
**8**	C	-0.255752	-0.222083	0.033669
**9**	C	0.62386	0.597486	-0.026374
**10**	C	0.57488	0.631298	0.056418
**11**	C	-0.04145	-0.063906	-0.022456
**12**	C	0.10906	0.11936	0.0103
**13**	C	-0.040985	0.050302	0.091287
**14**	C	0.059424	0.061599	0.002175
**15**	C	-0.128611	-0.166965	-0.038354
**16**	C	-0.280264	-0.285344	-0.00508
**17**	C	-0.031445	-0.065495	-0.03405
**18**	C	-0.286908	-0.366511	-0.079603
**19**	C	0.00416	-0.000923	-0.005083
**20**	C	0.047244	0.028196	-0.019048
**21**	C	-0.190786	-0.178	0.012786
**22**	C	-0.12466	-0.193633	-0.068973
**23**	C	-0.019627	-0.046982	-0.027355
**24**	C	0.010356	-0.021671	-0.032027
**25**	C	-0.11998	-0.117108	0.002872
**26**	C	-0.055679	-0.04109	0.014589
**27**	C	0.058154	0.054879	-0.003275
**28**	C	-0.019553	-0.018622	0.000931
**29**	C	-0.161274	-0.15621	0.005064

**30**	C	-0.139497	-0.129713	0.009784
**31**	Cl	-0.135275	-0.159915	-0.02464
**32**	C	-0.194116	-0.164206	0.02991
**33**	C	-0.069008	-0.019295	0.049713
**34**	C	-0.038422	-0.042641	-0.004219
**35**	C	-0.191228	-0.172977	0.018251
**36**	H	-0.045553	-0.026506	0.019047
**37**	H	0.105075	0.105492	0.000417
**38**	H	0.074649	0.114859	0.04021
**39**	H	0.105201	0.108842	0.003641
**40**	H	0.104569	0.111312	0.006743
**41**	H	0.095821	0.145488	0.049667
**42**	H	0.092036	0.08755	-0.004486
**43**	H	0.084617	0.064833	-0.019784
**44**	H	0.104047	0.134159	0.030112
**45**	H	0.11574	0.114812	-0.000928
**46**	H	0.183707	0.193554	0.009847
**47**	H	0.058165	0.03513	-0.023035
**48**	H	0.08179	0.186481	0.104691
**49**	H	0.138707	0.131466	-0.007241
**50**	H	0.107491	0.116152	0.008661
**51**	H	0.09448	0.118713	0.024233
**52**	H	0.130786	0.113337	-0.017449
**53**	H	0.110259	0.118296	0.008037
**54**	H	0.11604	0.118886	0.002846

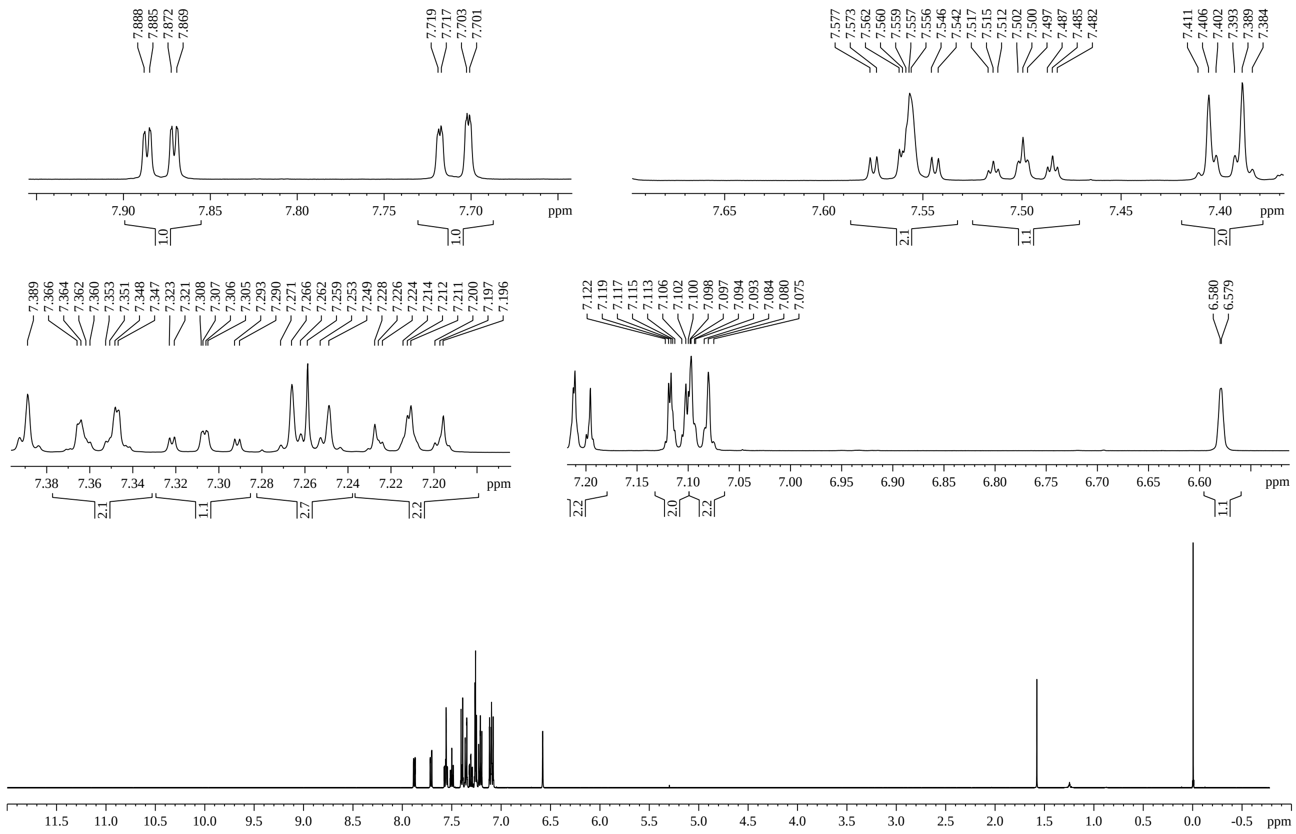

Figure S1. ^{1}H NMR spectrum (500 MHz, $CDCl_3$) of compound QCCP.

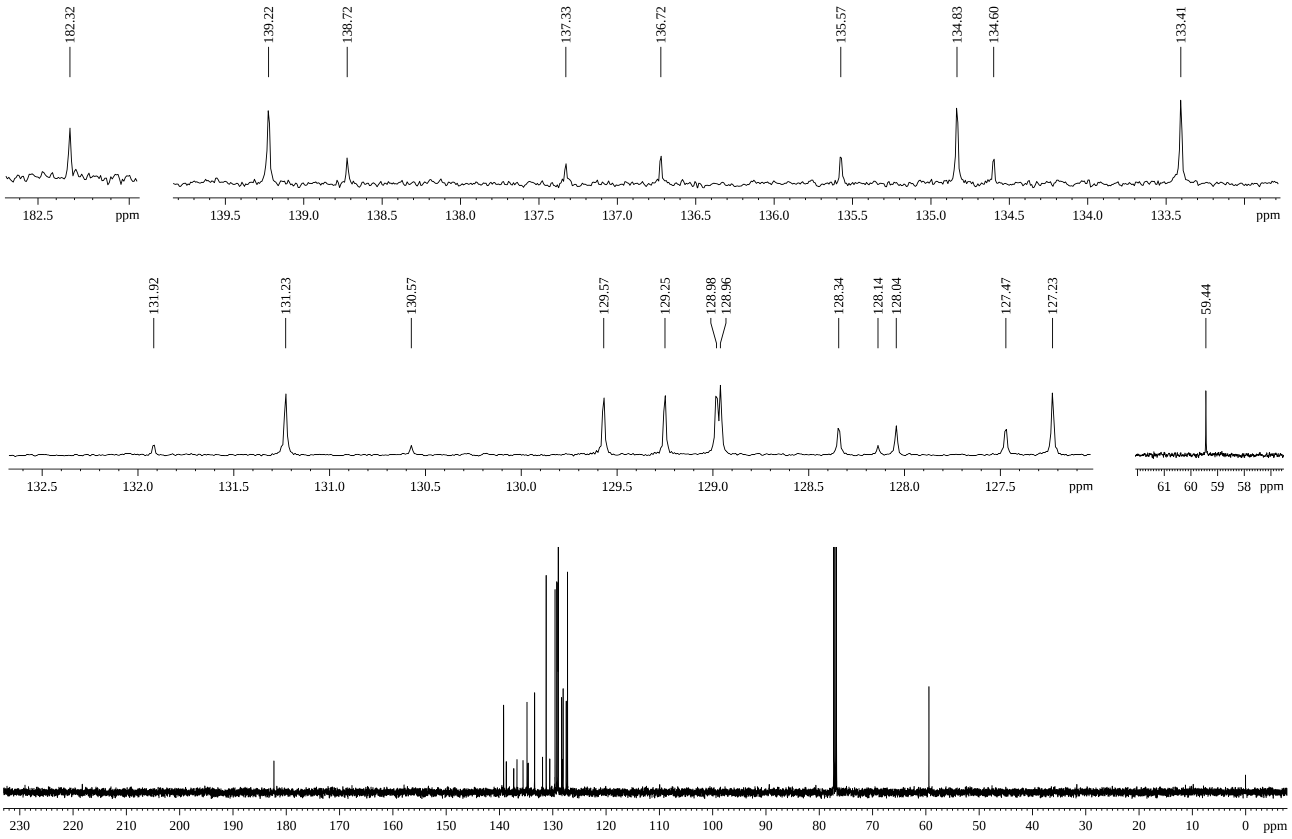

Figure S2. $^{13}C\{^{1}H\}$ NMR spectrum (126 MHz, $CDCl_3$) of compound QCCP.

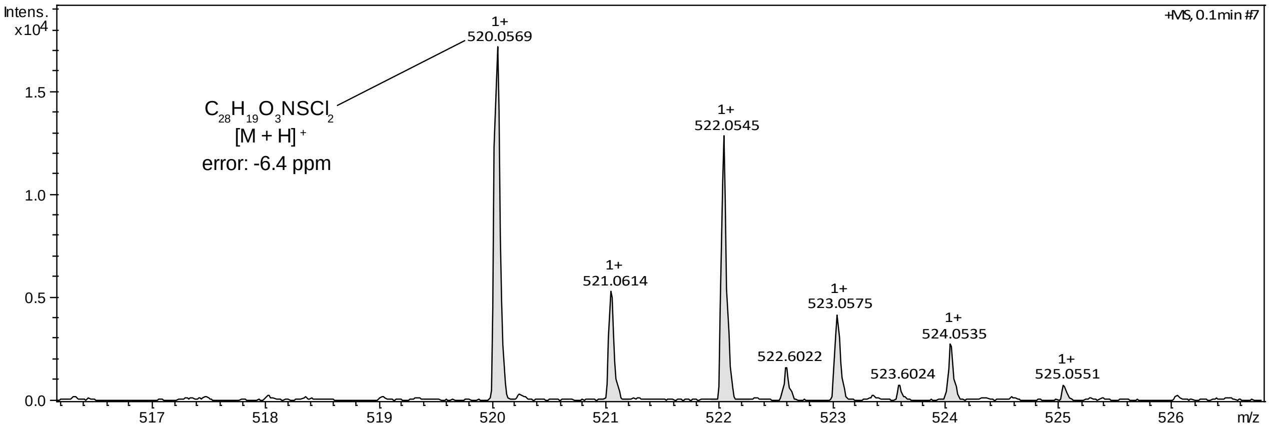

Figure S3. High-resolution mass spectrum of compound QCCP.

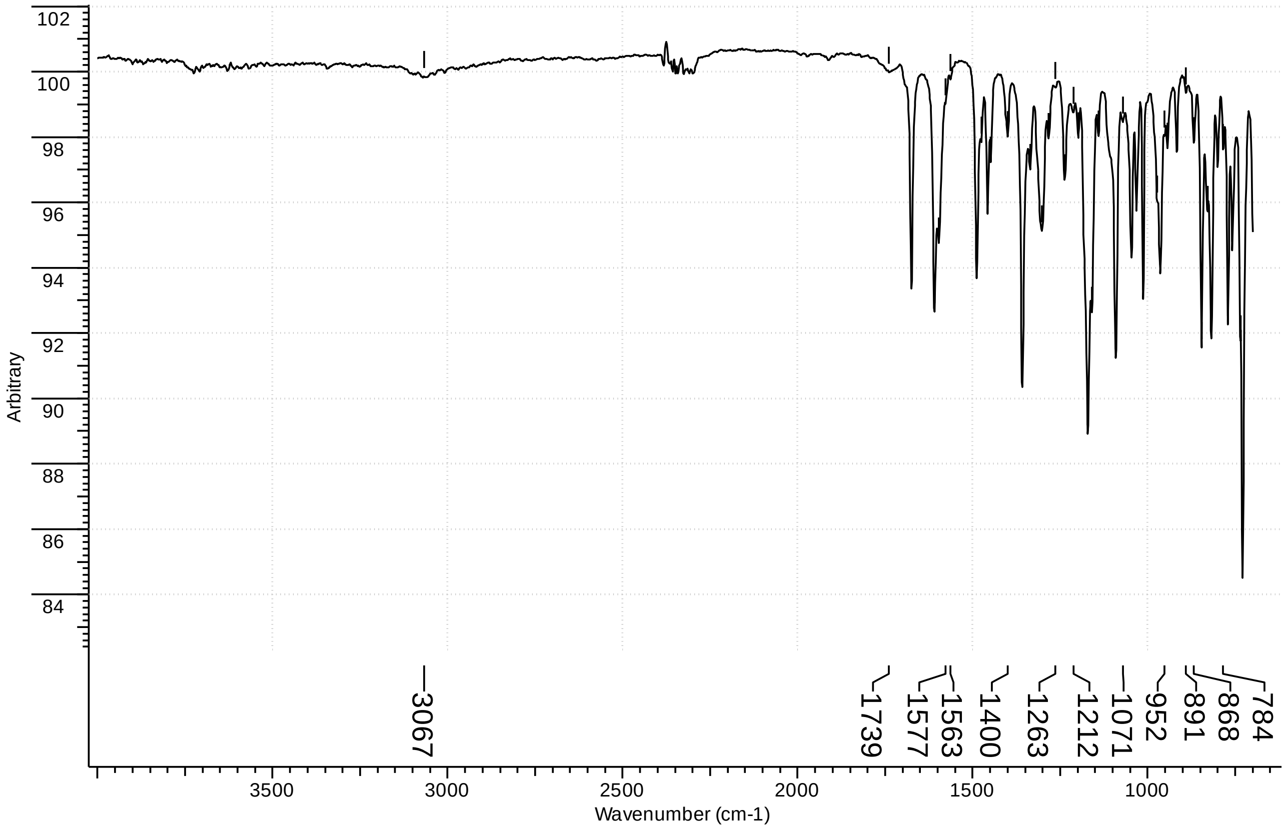

Figure S4. Infrared spectrum of compound QCCP.

Molecular modelling analysis

Figure S5 displays the frontier molecular orbitals, HOMO and LUMO, for QCCP in both isolated and embedded states. In both cases shown, the HOMO resides primarily on the B and C rings, encompassing the chlorine substituents and the nitrogen atom. The LUMO distribution, also consistent across both conditions, spans rings A and B, includes the chlorine atom on ring B, certain atoms on ring C, carbon C26 (part of ring D), and the sulfur atom. Additionally, Figure 5 illustrates the energy gap (ΔE), computed as the difference between the HOMO and LUMO energies. This gap exhibits a small decrease from 6.66 eV for the isolated molecule to 6.64 eV for the embedded molecule, a change attributable to crystalline environment polarization.

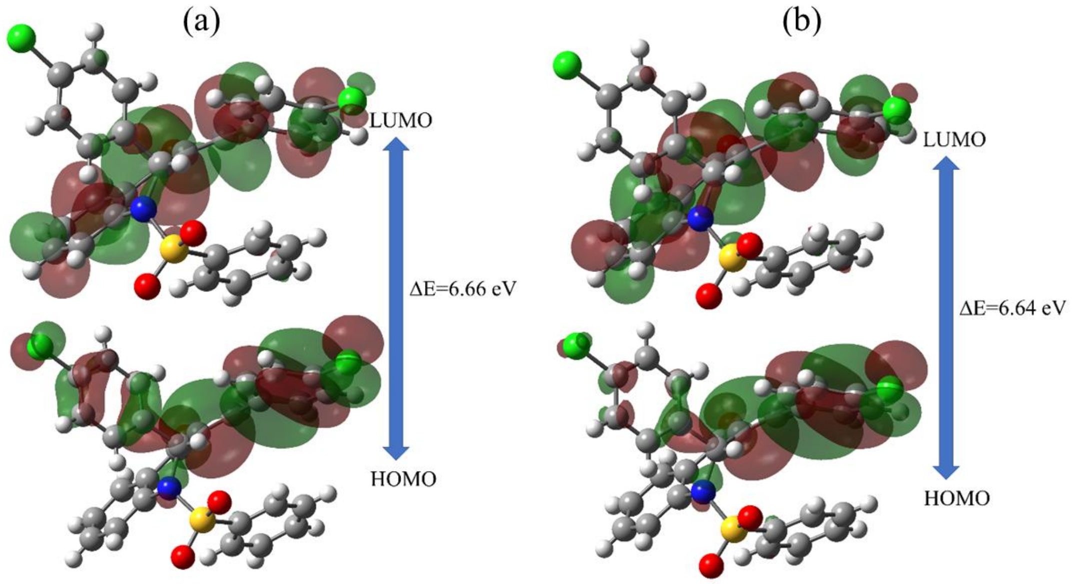

Figure S5. HOMO and LUMO frontiers orbitals and the gap energies for (a) isolated and (b) embedded QCCP molecules.

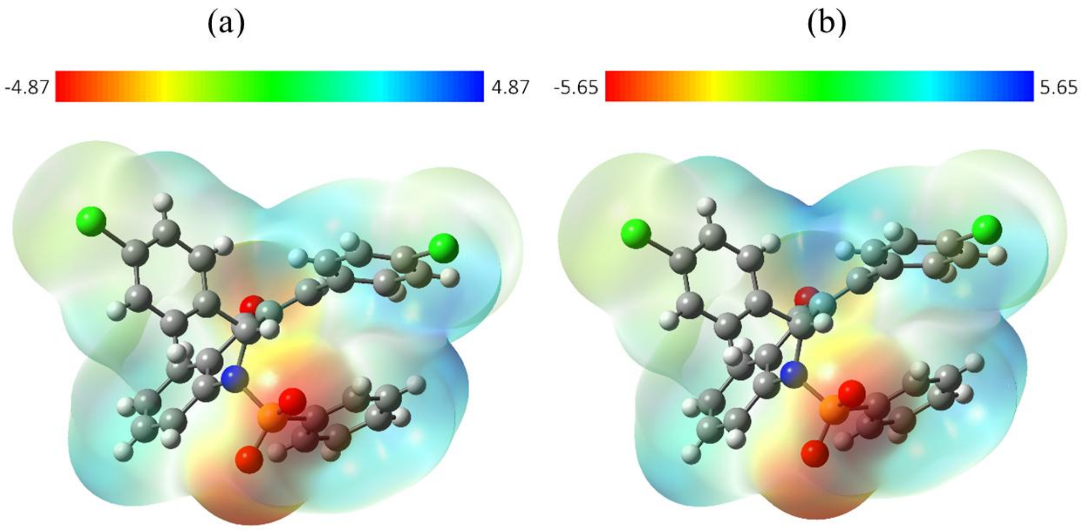

Figure S6. Molecular electrostatic potential (MEP) for both (a) isolated and (b) embedded molecules of QCCP.

Figure S6 (a) and (b) present the molecular electrostatic potential (MEP) for the isolated and embedded molecules. On the MEP map, blue regions correspond to positive potential, and red regions correspond to negative potential. Observation shows that the most negative electrostatic potential is situated near the three oxygen atoms. The most positive potential regions are found around the hydrogen atoms. Areas next to the chlorine atoms appear in green, signifying an intermediary part of the charge spectrum.

(1) Castro, M. R. C. de; Aragão, Â. Q.; Silva, C. C. da; Perez, C. N.; Queiroz, D. P. K.; Queiroz Júnior, L. H. K.; Barreto, S.; Moraes, M. O. de; Martins, F. T. Conformational Variability in Sulfonamide Chalcone Hybrids: Crystal Structure and Cytotoxicity. *J Braz Chem Soc* **2015**. https://doi.org/10.5935/0103-5053.20150341.

(2) d'Oliveira, G.; Moura, A.; de Moraes, M.; Perez, C.; Lião, L. Synthesis, Characterization and Evaluation of in Vitro Antitumor Activities of Novel Chalcone-Quinolinone Hybrid Compounds. *J Braz Chem Soc* **2018**. https://doi.org/10.21577/0103-5053.20180108.
